# Analysis of influencing factors for frailty in geriatric syndrome patients and the impact of frailty decompensation on major adverse events

**DOI:** 10.7717/peerj.21514

**Published:** 2026-07-14

**Authors:** Yirun Sun, Xiangqian Liu, Conggao Zhang, Ancai Wang, Deguo Wang

**Affiliations:** 1 Geriatrics Department, The Affiliated Chuzhou Hospital of Anhui Medical University, Chuzhou, Anhui Province, China; 2 Geriatrics Department, First Affiliated Hospital of Wanan Medical University, Wuhu, Anhui Province, China

**Keywords:** Geriatric syndrome, Frailty decompensation, Predictive model, Major adverse events

## Abstract

**Background:**

Frailty is a core manifestation of geriatric syndromes, and its decompensated state is associated with adverse outcomes, yet standardized assessment criteria are lacking.

**Methods:**

From January 2022 to June 2024, 538 patients with geriatric syndromes were prospectively enrolled. Frailty was assessed using the Fried phenotype. The optimal left ventricular ejection fraction (LVEF) cutoff (≤51.2%) for identifying frailty decompensation was determined *via* ROC curve analysis. Risk factors for frailty progression and the impact of decompensation on major adverse events (all-cause mortality, stroke, cardiovascular events, urinary incontinence) and hospitalization frequency were analyzed.

**Results:**

The prevalence of frailty was 44.05%. Independent risk factors for frailty progression included advanced age, malnutrition (low MNA-SF score and albumin), impaired cardiac function (low LVEF, NYHA class III–IV), anxiety/depression, and low hemoglobin. LVEF ≤ 51.2% effectively defined frailty decompensation (AUC = 0.781). Decompensation was an independent risk factor for urinary incontinence (OR = 4.113, *P* = 0.049) and for increased hospitalization frequency (IRR = 1.628, *P* = 0.003).

**Conclusions:**

This study identifies multisystem risk factors for frailty progression, proposes an exploratory, internally derived LVEF threshold for identifying frailty decompensation, which should be considered hypothesis-generating and requires external validation before clinical application, and confirms that decompensation significantly increases the risk of urinary incontinence and healthcare burden.

## Introduction

Geriatric syndrome is a collective term for various complex disorders, which refers to a group of clinical manifestations in older adults caused by multiple pathological processes or predisposing factors. This type of condition severely impairs the functional capacity of the elderly, significantly reduces their quality of life, and markedly shortens their life expectancy. Common geriatric syndromes include dementia, dizziness, delirium, falls, insomnia, pressure ulcers, pain, urinary incontinence, constipation, frailty, sarcopenia, dysphagia, polypharmacy, among others. Among these, frailty syndrome (FS) is a geriatric condition characterized by diminished physiological reserve and impaired stress resistance, resulting from the cumulative decline across multiple organ systems (Damluji et al., 2021b). This clinical state renders older adults particularly susceptible to adverse outcomes-including falls, hospitalization, disability, and mortality-even in response to minor stressors such as acute illness or changes in the living environment  (Barbosa da Silva et al., 2020). Originally conceptualized as a phenotypic syndrome by Fried et al. (2001), frailty is operationally defined by five specific criteria: unintentional weight loss, self-reported fatigue, reduced grip strength, slow gait speed, and low physical activity, with the presence of three or more indicators confirming frailty. Epidemiological data indicate that the prevalence of frailty reaches up to 30% among individuals aged 80 years and older, with a higher incidence observed in women (Rodriguez-Manas & Fried, 2015). Importantly, frailty is considered a dynamic and potentially reversible condition, underscoring the importance of early identification of its contributing factors for timely intervention and prevention (Pandey, Kitzman & Reeves, 2019).

The pathogenesis of frailty is a highly intricate process, arising from the complex and dynamic interplay among a diverse array of factors. These encompass biological mechanisms, typified by the phenomenon of “inflammaging”; sociobehavioral elements, as exemplified by a lack of adequate family or social support; metabolic issues, with malnutrition being a prominent case; and cardiovascular processes (Thillainadesan, Scott & Le Couteur, 2020; Li et al., 2023; Muszalik et al., 2019; Damluji et al., 2021b). Frailty decompensation-an acute exacerbation of physiological vulnerability triggered by external stressors such as infection or metabolic disturbances-represents a critical clinical transition, frequently resulting in rapid functional decline (Damluji et al., 2021a). In contrast to compensated frailty, decompensation is characterized by a loss of homeostatic resilience, wherein minor stressors can precipitate progressive and systemic organ dysfunction (Zeng et al., 2024; Guidet et al., 2020). Despite its clinical relevance, there is a paucity of studies directly comparing the incidence of adverse outcomes between compensated and decompensated frailty subgroups, and consensus on standardized diagnostic criteria for frailty decompensation remains lacking.

Accurate diagnosis of frailty and its decompensated state requires comprehensive, integrated assessments. Phenotypic screening tools such as the FRAIL scale and nutritional evaluation instruments like the Mini Nutritional Assessment Short-Form (MNA-SF) are valuable for initial identification of at-risk individuals (Mishra et al., 2024; Álvarez-Millán et al., 2023). Furthermore, the integration of frailty scales with objective biomarkers-such as interleukin-6-has been shown to improve prognostic accuracy (Aydin et al., 2025; Soysal et al., 2016; Picca et al., 2022). Emerging evidence suggests that cardiac function, particularly left ventricular ejection fraction (LVEF), may serve as a key determinant in the progression to frailty decompensation (Rizza et al., 2021; Huang et al., 2025). However, the utility of LVEF as a predictive cutoff for adverse clinical events remains insufficiently investigated.

This study aims to: (1) identify independent risk factors associated with frailty in patients presenting with geriatric syndromes; (2) develop a predictive model for frailty progression incorporating left ventricular ejection fraction (LVEF); (3) define frailty decompensation using an LVEF threshold of ≤51.2% (determined *via* receiver operating characteristic [ROC] curve analysis) and the risk factors for major adverse events-including all-cause mortality, stroke, cardiovascular events, and urinary incontinence-between compensated (LVEF > 51.2%) and decompensated frailty subgroups were analyzed; and (4) investigate the association between frailty decompensation and hospitalization frequency. The findings of this study are expected to contribute to the development of targeted interventions aimed at reducing adverse clinical outcomes in this vulnerable patient population.

## Materials and Methods

### Study design and participants

A prospective cohort study was conducted at The Affiliated Chuzhou Hospital of Anhui Medical University. Baseline enrollment was carried out consecutively from January 2022 to June 2024, during which 538 patients diagnosed with geriatric syndromes were recruited from the Departments of Geriatrics and Cardiovascular Medicine. A preliminary screening was conducted through the electronic medical record system, identifying 983 patients aged ≥60 years who were continuously seen during this period. Subsequently, a stepwise screening was performed based on stringent inclusion and exclusion criteria: (1) First, 87 patients with a visit age of <60 years were excluded; (2) following independent review by two senior attending physicians, 63 patients who did not meet the diagnostic criteria for geriatric syndromes were excluded; (3) according to the exclusion criteria, further exclusions were made for patients with concurrent malignancies/acute trauma (*n* = 38), those with cognitive impairment/severe psychiatric disorders (*n* = 41), patients who were completely functionally dependent or bedridden (*n* = 29), and those with missing core clinical data (*e.g.*, LVEF, MNA-SF score, serum albumin) (*n* = 51); (4) 36 patients refused to sign the informed consent form. Ultimately, 538 patients were included in the final analysis cohort (the screening process is detailed in Fig. S1). Inclusion criteria were as follows: (1) age ≥60 years; (2) presence of clinical symptoms, electrocardiographic findings, and echocardiographic results consistent with established diagnostic guidelines for geriatric syndromes. Exclusion criteria included: (1) concurrent diagnosis of malignant tumors, hematological disorders, or acute trauma; (2) cognitive impairment, dementia, or severe psychiatric illness; (3) complete functional dependence or bedridden status; (4) significant hearing loss or communication barriers that would impede study participation. The study protocol was reviewed and approved by the Ethics Committee of The Affiliated Chuzhou Hospital of Anhui Medical University (Approval No.: CZYY-2022-015) and the study was conducted in full accordance with the ethical principles of the Declaration of Helsinki. Written informed consent was obtained from all participants or their legally authorized representatives prior to study initiation.

In this study, geriatric syndrome refers to a group of clinical syndromes that are commonly seen in older adults, caused by multiple etiologies, and characterized by specific clinical manifestations. Included patients were required to meet at least one of the following syndromes with an established diagnosis based on relevant guidelines or consensus: frailty (according to the Fried phenotype criteria), sarcopenia (according to the Asian Working Group for Sarcopenia consensus), falls (≥2 unintentional falls within the past year), urinary incontinence (according to the International Consultation on Incontinence criteria), or late-life depression (according to the DSM-5 criteria). All diagnoses were independently reviewed and confirmed by two senior attending physicians. In cases of disagreement, consensus was reached through discussion or adjudication by a third chief physician to ensure consistent application of the diagnostic criteria.

### Variables and assessment methods

#### General information

The collected data encompassed demographic and clinical variables including age, sex, smoking and alcohol consumption habits, body mass index (BMI), New York Heart Association (NYHA) functional classification, Mini Nutritional Assessment-Short Form (MNA-SF) score, and the presence of comorbid conditions such as type 2 diabetes mellitus, hypertension, chronic obstructive pulmonary disease (COPD), cerebrovascular disease, and anxiety or depression. The presence of anxiety and depressive symptoms was systematically assessed using the Hamilton Depression Rating Scale (HAMD).

#### Laboratory and imaging indicators

Main exposure indicator: Left ventricular ejection fraction (LVEF) was measured using echocardiography according to the apical biplane Simpson method, as recommended by the 2015 guidelines from the American Society of Echocardiography and the European Association of Cardiovascular Imaging. Specifically, the measurement protocol strictly followed Section 3.2 of these guidelines, which details standardized procedures for left ventricular volume quantification. This involved acquiring two orthogonal apical views-the four-chamber and two-chamber views-with high-quality imaging to ensure complete visualization of the endocardial borders throughout the entire cardiac cycle.

All echocardiographic assessments were conducted by two certified echocardiographers with 8 and 10 years of specialized experience in geriatric cardiac imaging, respectively, both of whom were accredited with Level 3 certification by the Chinese Society of Echocardiography. To ensure measurement consistency and reliability, intra-observer and inter-observer variability analyses were performed on a randomly selected subset of 50 patients. The intraclass correlation coefficients (ICC) for LVEF measurements were 0.93 for intra-observer agreement and 0.89 for inter-observer agreement, indicating excellent reproducibility and surpassing the clinically acceptable threshold of 0.85 as specified in the guidelines.

End-diastolic and end-systolic endocardial borders were manually traced by trained operators, and image analysis was conducted using the GE Vivid E95 ultrasound system software (Version 2023.1). Left ventricular ejection fraction (LVEF) was calculated using the standard formula: (end-diastolic volume − end-systolic volume)/end-diastolic volume ×100%. To ensure measurement accuracy, automated quality assurance protocols, as outlined in the guidelines (Picca et al., 2022), were implemented to exclude datasets with suboptimal endocardial delineation or significant ventricular foreshortening (defined as ≤15% of ventricular length).

#### Frailty assessment

Frailty was defined according to the Fried phenotype criteria (Fried et al., 2001), which consist of five components: (1) unintentional weight loss (>4.5 kg within 1 year); (2) self-reported fatigue; (3) reduced physical activity; (4) decreased grip strength (adjusted for gender and body size); and (5) slow walking speed (adjusted for gender and height). Individuals meeting ≥3 of these criteria were categorized as frail, while those with fewer than 3 were classified as non-frail. The Fried frailty phenotype was selected in this study for the following reasons: (a) it is the most widely cited and classical operational definition in frailty research, facilitating comparison of our findings with a large body of existing literature; (b) it emphasizes objectively measurable physiological components, which reflect declines in multisystem physiological reserve and align well with the study’s aim of exploring associations with cardiac and other organ functions; and (c) compared with cumulative deficit models (such as the Frailty Index), the phenotype approach is more concise and clinically feasible, making it well suited for implementation in our study cohort.

Frailty progression was operationally defined as individuals who were classified as non-frail at baseline and subsequently transitioned to meeting the Fried frailty criteria (≥3 components) during the follow-up period, or as individuals who were classified as frail at baseline and subsequently developed one of the new Fried frailty phenotypes during follow-up, meeting the Fried frailty criteria (≥3 components).

#### Follow-up and outcome definition

Follow-up was conducted for a median duration of 24 months (interquartile range: 19–32 months) through scheduled outpatient visits every 3 months and monthly telephone interviews. All five Fried components were comprehensively reassessed at each scheduled outpatient visit, which occurred every 3 months. In the event of an unscheduled visit, an additional frailty assessment was performed; however, the time-to-event analysis primarily used the date of the first scheduled visit at which the progression criteria were met as the event time. Telephone follow-ups were not used for complete Fried phenotype ascertainment; their purpose was limited to confirming vital status, adverse events, and follow-up adherence. The event time for frailty progression was defined as the date of the first outpatient visit at which the patient met the progression criteria. Major adverse events, serving as study endpoints, were defined as follows: (1) all-cause mortality: death from any cause, verified by medical documentation or family reports; (2) stroke: an acute cerebrovascular event confirmed by cranial computed tomography or magnetic resonance imaging; (3) cardiovascular events: including acute myocardial infarction, hospitalization for heart failure, or arrhythmia requiring clinical intervention, all diagnosed by attending physicians; and (4) urinary incontinence: involuntary urine leakage occurring at least once per week, as reported by patients or caregivers and confirmed by nursing staff. The enrollment date was used as the time origin in the Cox model, with follow-up continuing until the occurrence of the outcome event or censoring at the last follow-up. The endpoint event for the Cox regression model of frailty progression is frailty progression, with detailed reference to the operational definition of frailty progression.

#### Operational definition of frailty decompensation

A patient was classified as having experienced frailty decompensation if any of the following occurred within the follow-up period: (1) Unplanned hospitalization due to progression of the underlying disease or new-onset complications; (2) new or acutely worsened organ dysfunction, including heart failure (clinical diagnosis per Framingham criteria) or acute kidney injury (serum creatinine increase ≥0.3 mg/dL or ≥1.5-fold from baseline); (3) New loss of at least one basic activity of daily living (ADL).

### Statistical analysis

Data were analyzed using SPSS version 26.0. Continuous variables were summarized as mean ± standard deviation for normally distributed data (assessed using the Shapiro–Wilk test) or as median (interquartile range) for non-normally distributed data. Categorical variables were presented as frequencies (percentages). Group comparisons were performed using independent samples *t*-tests for normally distributed data, Mann–Whitney *U* tests for non-normal data, and Chi-square tests for categorical variables.

Univariate Cox regression analysis was used to identify potential predictors of frailty progression (*P* < 0.05). Multivariable Cox regression models were subsequently adjusted for relevant confounding variables, with hazard ratios (HR) and corresponding 95% confidence intervals (CI) reported. Receiver operating characteristic (ROC) curve analysis was employed to determine the optimal LVEF threshold for identifying frailty decompensation, with the area under the curve (AUC), sensitivity, and specificity calculated to evaluate diagnostic performance. The binary reference outcome for the ROC analysis was the clinical event of frailty decompensation, which was independently defined according to the operational definition of frailty decompensation. The LVEF cutoff was derived post hoc from the same dataset in an exploratory manner.

The association between hospitalization frequency and frailty decompensation was evaluated using negative binomial regression analysis. Variables were selected based on clinical relevance and univariate analyses, with potential collinearity taken into account. Missing data, accounting for less than 5% of values across all variables, were handled using multiple imputation *via* the mice package in R. Five complete datasets were created through 10 iterations each. Different imputation methods were applied based on variable types: continuous variables were imputed using predictive mean matching (PMM), binary variables using logistic regression, and categorical variables with more than two levels using polytomous logistic regression. Sensitivity analyses were conducted by excluding imputed data to confirm the robustness of the findings. A *P*-value < 0.05 was considered statistically significant.

## Results

### Baseline characteristics and frailty prevalence

Among the 538 enrolled patients with geriatric syndromes, 237 (44.05%) were classified as frail according to the Fried phenotype criteria, highlighting a high prevalence of frailty in this population-consistent with the study’s primary objective of identifying frailty-related patterns among patients with geriatric syndromes. A comparative analysis between frail and non-frail groups revealed significant differences in several key clinical indicators that are central to the study’s aim of exploring factors associated with frailty. Frail patients were significantly older (median, 76.5 *vs.* 72.0, *P* < 0.001), which aligns with the well-established association between aging and physiological decline-a core mechanism hypothesized to contribute to frailty. Frail patients had a significantly lower BMI than the non-frail group (median, 21.4 *vs.* 22.5, *P* < 0.001), even though both values were within the normal range. They also demonstrated lower Mini Nutritional Assessment-Short Form (MNA-SF) scores (median, 11.0 *vs.* 12.0, *P* < 0.001) and lower serum albumin levels (median, 40.4 *vs.* 42.7 g/L, *P* < 0.001), supporting the study’s investigation into malnutrition as a potential contributor to frailty. Furthermore, frail patients exhibited reduced left ventricular ejection fraction (LVEF) (median, 46.4% *vs.* 49.7%, *P* < 0.001), a higher proportion of New York Heart Association (NYHA) class III–IV heart failure (69.6% *vs.* 31.2%, *P* < 0.001), and a greater prevalence of anxiety or depression (28.7% *vs.* 13.3%, *P* < 0.001)—all variables that were specifically examined in the study to assess the impact of cardiac and psychological factors on frailty. Hemoglobin levels were significantly lower in frail patients (mean, 110.39 ± 15.31 *vs.* 119.98 ± 14.27, *P* < 0.001), whereas serum creatinine levels (mean, 116.58 ± 16.85 *vs.* 112.46 ± 19.13, *P* = 0.01), the prevalence of cerebrovascular disease (28.7% *vs.* 16.6%, *P* < 0.001), and the prevalence of hypertension (61.6% *vs.* 52.5%, *P* = 0.034) were significantly higher in the frail group than in the non-frail group. Notably, the proportion of smokers was paradoxically higher in the non-frail group compared with the frail group (38.2% *vs.* 28.7%, *P* = 0.021). No significant differences were found in terms of gender, alcohol consumption, COPD, and Type 2 Diabetes, suggesting that these factors may not be primary contributors to frailty in this patient cohort (Table 1).

**Table 1:** Baseline characteristics and multivariable Cox regression analysis of frailty risk factors (*N* = 538).

Variables	Non-frail group (*n* = 301)	Frail group (*n* = 237)	*P*	HR (95% CI)	*P*
Age (years)	72.0 (60–91)	76.5 (63–91)	<0.001	1.112 (1.084–1.142)	<0.001
BMI (kg/m^2^)	22.5 (17.5–30.6)	21.4 (17.5–28.1)	<0.001	0.924 (0.874–0.977)	0.005
MNA-SF score	12.0 (6–14)	11.0 (4–14)	<0.001	0.799 (0.748–0.855)	<0.001
Albumin (g/L)	42.7 (28–52)	40.4 (28–52)	<0.001	0.953 (0.93–0.976)	<0.001
LVEF (%)	49.7 (33.6–69.5)	46.4 (32.7–68.2)	<0.001	0.956 (0.935–0.977)	<0.001
Hemoglobin (g/L)	119.98 ± 14.27	110.39 ± 15.31	<0.001	0.98 (0.97–0.989)	<0.001
Serum creatinine (µmol/L)	112.46 ± 19.13	116.58 ± 16.85	0.01	1.001 (0.993–1.009)	0.820
Gender			0.911		
Female, *n* (%)	131 (43.5%)	102 (43.0%)			
Male, *n* (%)	170 (56.5%)	135 (57.0%)			
Smoking, *n* (%)	115 (38.2%)	68 (28.7%)	0.021	0.823 (0.602–1.125)	0.221
Alcohol, *n* (%)	62 (20.6%)	64 (27.0%)	0.082		
NYHA class, *n* (%)			<0.001		
I	112 (37.2%)	24 (10.1%)		Ref	
II	95 (31.6%)	48 (20.3%)		1.816 (0.964–3.421)	0.065
III	59 (19.6%)	78 (32.9%)		5.185 (2.869–9.37)	<0.001
IV	35 (11.6%)	87 (36.7%)		5.517 (3.024–10.066)	<0.001
Hypertension, *n* (%)	158 (52.5%)	146 (61.6%)	0.034	1.15 (0.855–1.547)	0.355
Type 2 diabetes, *n* (%)	79 (26.2%)	74 (31.2%)	0.204		
COPD, *n* (%)	68 (22.6%)	58 (24.5%)	0.609		
Cerebrovascular disease	50 (16.6%)	68 (28.7%)	0.001	0.917 (0.666–1.264)	0.596
Anxiety or depression	40 (13.3%)	68 (28.7%)	<0.001	2.293 (1.663–3.162)	<0.001

### Independent risk factors for frailty progression and predictive model performance

Among the 538 patients with geriatric syndromes, 207 individuals experienced the progression of frailty, all of whom were from the baseline frailty group. Variables showing statistically significant differences between groups (*P* < 0.05) in univariate analysis were included in the multivariable Cox proportional hazards model. Nine independent factors significantly associated with frailty progression were ultimately identified (all *P* < 0.05). Specifically, increasing age (HR = 1.112, 95% CI [1.084–1.142]), NYHA functional class III–IV (HR = 5.185–5.517), and the presence of anxiety/depression (HR = 2.293, 95% CI [1.663–3.162]) significantly increased the risk of frailty progression. Conversely, higher MNA-SF scores (HR = 0.799, 95% CI [0.748–0.855]), left ventricular ejection fraction (LVEF) (HR = 0.956, 95% CI [0.935–0.977]), albumin levels (HR = 0.953, 95% CI [0.930–0.976]), hemoglobin levels (HR = 0.980, 95% CI [0.970–0.989]), and body mass index (BMI) (HR = 0.924, 95% CI [0.874–0.977]) served as protective factors (Table 1).

**Figure 1:**
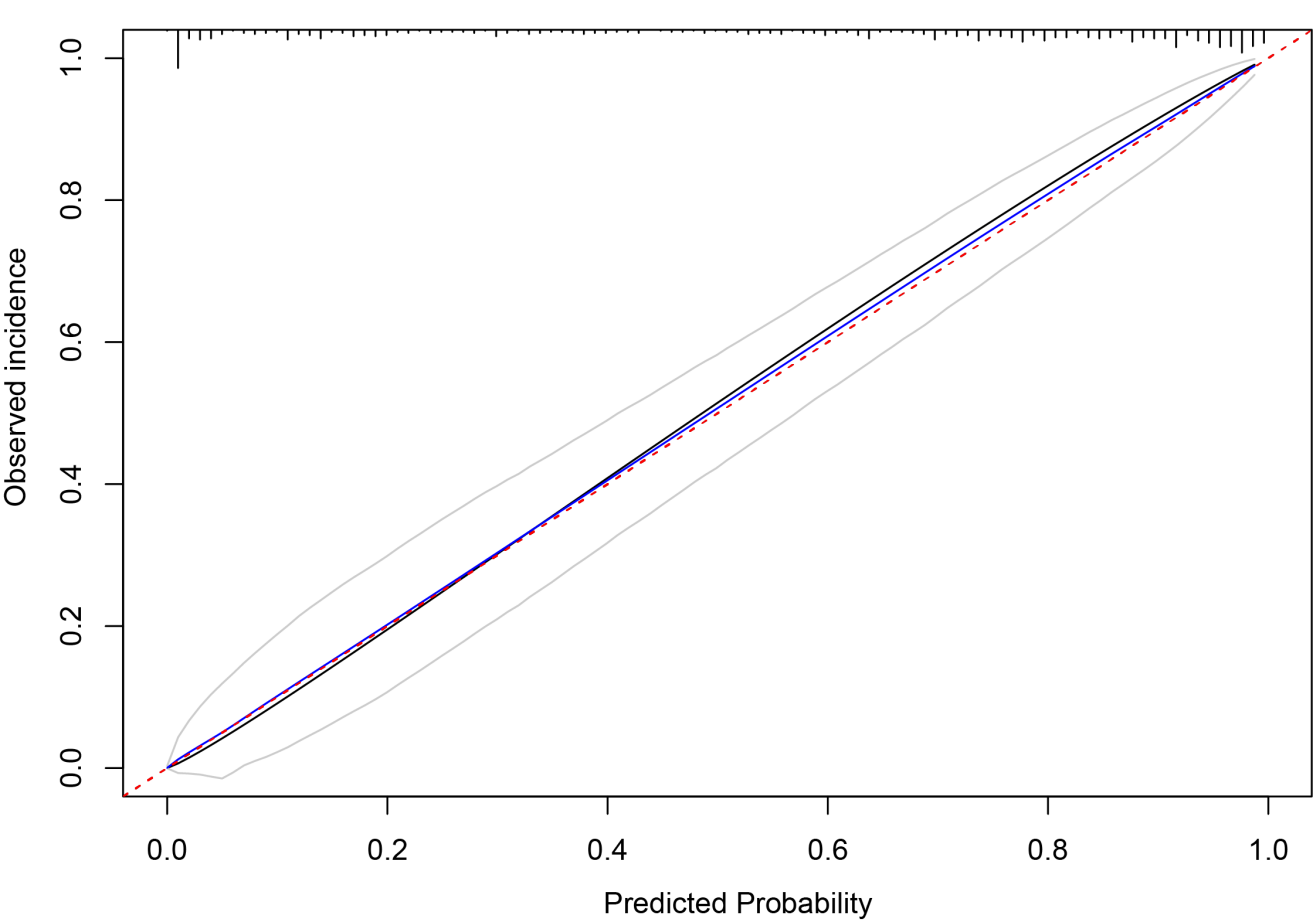
Calibration curve for the predictive model of frailty progression. The plot demonstrates the agreement between predicted probabilities of frailty progression (*x*-axis) and the observed event rates (*y*-axis) across the study cohort. The diagonal line represents perfect calibration. The close alignment of the observed incidence (represented by colored lines/dots, each indicating a specific iteration or subgroup from the multiple imputation analysis) to the ideal line indicates good model calibration.

The predictive model for frailty progression constructed based on the above factors demonstrated excellent performance. The mean C-index after multiple imputation was 0.84 (range: 0.839–0.841), indicating strong discriminative ability. Calibration curve analysis revealed good agreement between predicted probabilities and observed event rates across ten risk subgroups, with an average calibration deviation of 0.04, suggesting favorable model calibration. Furthermore, the model achieved an R^2^ of 0.475 and a Brier score of 0.1414, further confirming its robustness in predicting frailty progression within this study population (Fig. 1).

To verify the robustness of the results, a sensitivity analysis was conducted by excluding 52 patients with missing data (*n* = 486). The results demonstrated high consistency with those from the full analysis dataset following multiple imputation (Table S1). The dataset yielded a C-index (Concordance index) of 0.835, with a 95% CI [0.808–0.863]. This finding confirms the stability and reliability of the identified risk factors.

### LVEF cutoff for frailty decompensation

**Figure 2:**
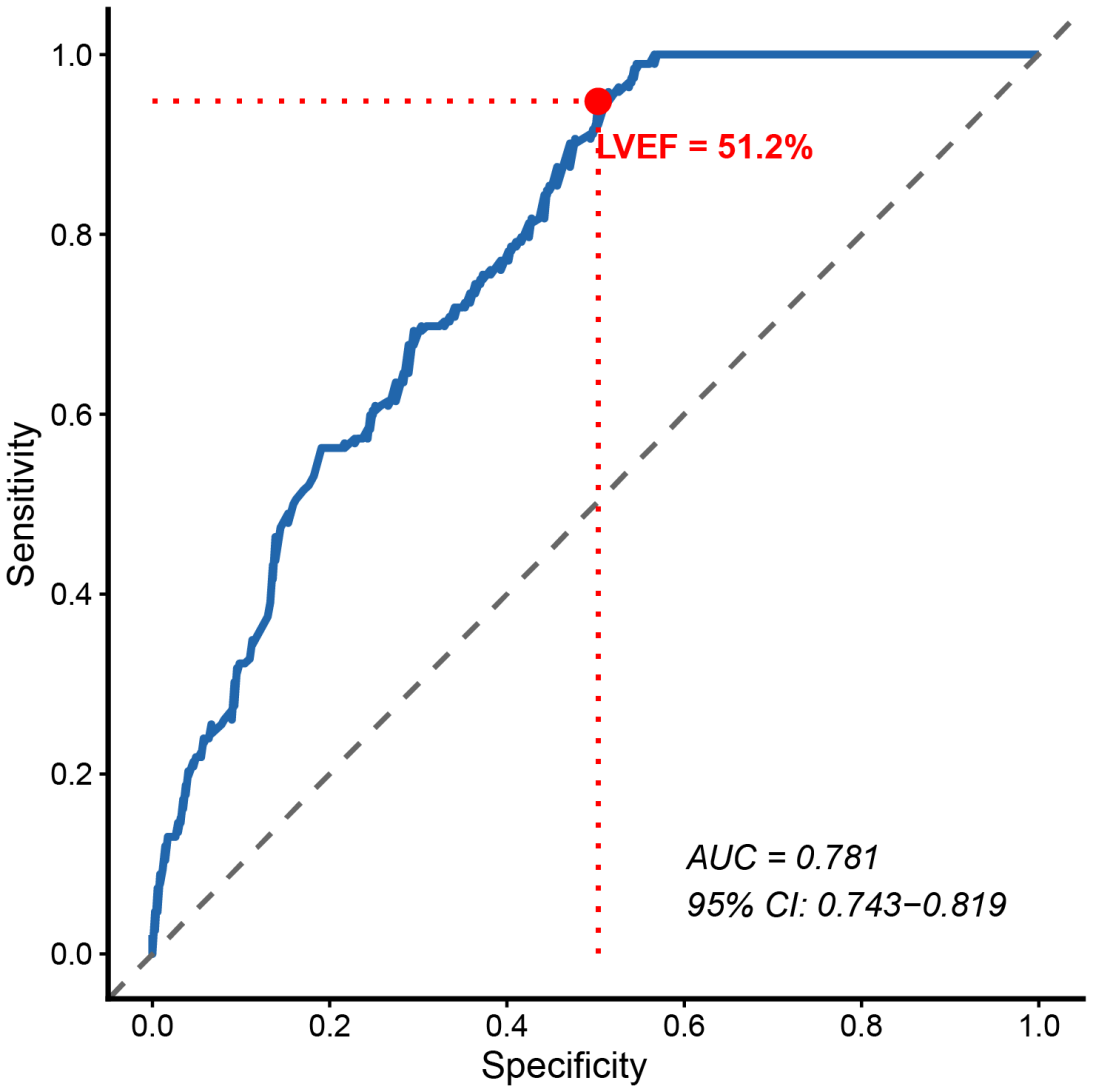
ROC curve for LVEF predicting frailty decompensation. The ROC curve (blue line) illustrates the predictive performance of left ventricular ejection fraction (LVEF) for frailty decompensation, with an area under the curve (AUC) of 0.781. The diagonal gray line represents the reference line (AUC = 0.5, indicating no discriminative ability). The optimal threshold is indicated by the black dot (LVEF = 51.2%), corresponding to a sensitivity of 0.948 (ability to correctly identify decompensated cases) and a specificity of 0.497 (ability to correctly identify compensated cases). This threshold maximizes the Youden index (0.445), thereby supporting its utility in clinical decision-making.

A primary objective of the study was to define frailty decompensation using an evidence-based left ventricular ejection fraction (LVEF) threshold. The ROC analysis was performed using frailty decompensation, defined according to the operational definition, as the binary reference outcome. Receiver Operating Characteristic (ROC) curve analysis of LVEF for predicting decompensation demonstrated strong diagnostic performance, with an area under the curve (AUC) of 0.781 (95% CI [0.743–0.819], *P* < 0.001). The optimal cutoff value was determined to be 51.2% based on maximization of the Youden index, yielding a sensitivity of 0.948 and specificity of 0.497 (Youden index = 0.445). This threshold may provide a useful reference for risk stratification, pending external validation. In contrast to previous studies that lacked specific LVEF cutoffs for assessing frailty, this value facilitates reproducible risk stratification-an essential step in translating research findings into potential clinical applications (Table 2, Fig. 2).

**Table 2:** Predictive value of LVEF for frailty decompensation (ROC curve analysis).

Index	Value
AUC	0.781
SE	0.019
95% CI	0.743–0.819
*P*	<0.001
Optimal threshold	51.2%
Sensitivity	0.948
Specificity	0.497
Youden index	0.445

**Figure 3:**
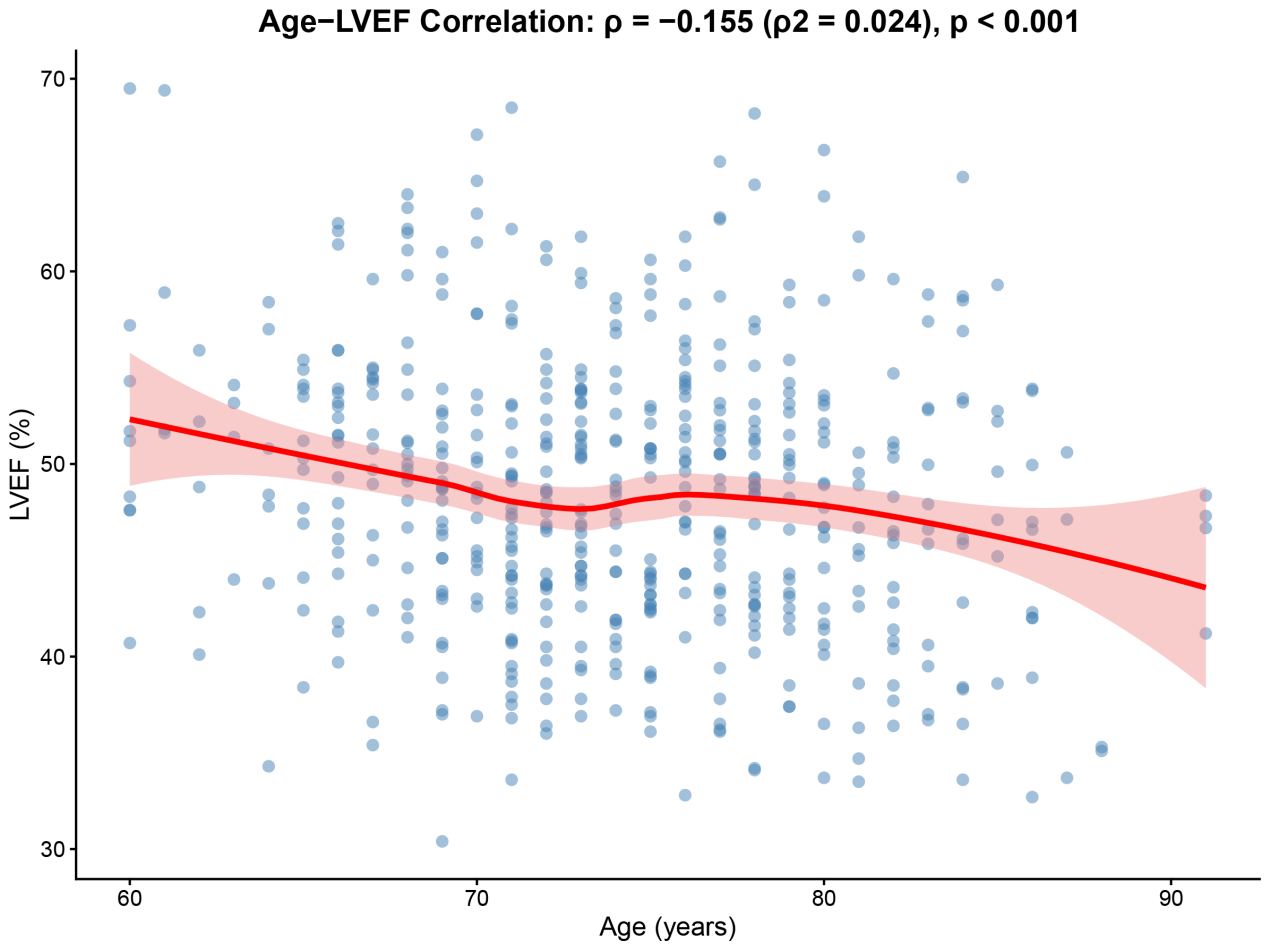
Scatter plot showing the correlation between age and Left Ventricular Ejection Fraction (LVEF) in the overall study population. A weak but statistically significant negative correlation was observed (Spearman’s *ρ* =  − 0.155, *P* < 0.001), with age explaining only 2.4% (*ρ*^2^ = 0.024) of the variance in LVEF.

Because age may influence LVEF, a correlation analysis between age and LVEF was performed using Spearman’s rank correlation. The results showed a correlation coefficient of *ρ* = −0.155 with statistical significance (*P* < 0.001), and an *ρ*^2^ of 0.024, indicating that age explained only 2.4% of the total variance in LVEF. The scatter plot (Fig. 3) visually illustrates this weak association.

To further assess age-related heterogeneity in this relationship, stratified analyses by age group (60–74 years, 75–84 years, and ≥85 years) were conducted (Fig. S2). In the 60–74-year group, a weak but statistically significant negative correlation was observed (*ρ* = −0.176, *P* = 0.003). In the 75–84-year group, the correlation was very weak and not statistically significant (*ρ* = −0.075, *P* = 0.265). In the ≥85-year group, a weak negative correlation was also observed but did not reach statistical significance (*ρ* = −0.287, *P* = 0.154), likely due to the limited sample size (*N* = 26). Across age strata, the proportion of LVEF variance explained by age (*ρ*^2^) ranged from 3.1% to 8.3%, further supporting the notion that age has a limited clinical impact on LVEF.

### Adverse events in compensated *vs.* decompensated groups

Among 237 frail patients, 45 were in the compensated group, and 192 were in the decompensated group. A comparison of baseline characteristics between the two groups revealed that the decompensated group had significantly lower hemoglobin levels than the compensated group (109.45 ± 14.968 g/L *vs.* 114.17 ± 16.249 g/L, *P* = 0.050). Additionally, the median left ventricular ejection fraction (LVEF) was significantly lower in the decompensated group compared to the compensated group (43.90% *vs.* 54.05%, *P* < 0.001). Furthermore, the proportion of patients classified as New York Heart Association (NYHA) functional class III–IV was markedly higher in the decompensated group (34.9% *vs.* 24.4% and 41.7% *vs.* 15.6%, respectively), while the proportions of patients in NYHA class I and II were significantly lower (*P* < 0.001) (Table 3).

**Table 3:** Comparison of baseline characteristics between the compensated and decompensated groups.

Indicator	Compensated group (*n* = 45)	Decompensated group (*n* = 192)	*P*
Age (years)	75.69 ± 5.581	76.79 ± 5.585	0.196
Hemoglobin (g/L)	114.17 ± 16.249	109.45 ± 14.968	0.050
Serum creatinine (µmol/L)	115.86 ± 15.524	116.75 ± 17.199	0.776
BMI (kg/m^2^)	21.5 (17.5–26.7)	21.3 (17.5–28.1)	0.349
MNA-SF score	12 (4–14)	11 (5–14)	0.507
Albumin (g/L)	41.8 (28–52)	40.25 (28–52)	0.264
LVEF (%)	54.05 (52.58–68.20)	43.90 (32.70–52.22)	<0.001
Sex, *n* (%)			0.260
Female	16 (35.6)	86 (44.8)	
Male	29 (64.4)	106 (55.2)	
Smoking, *n* (%)	12 (26.7)	56 (29.2)	0.739
Alcohol, *n* (%)	15 (33.3)	49 (25.5)	0.288
NYHA class, *n* (%)			<0.001
I	10 (22.2)	14 (7.3)	
II	17 (37.8)	31 (16.1)	
III	11 (24.4)	67 (34.9)	
IV	7 (15.6)	80 (41.7)	
Hypertension, *n* (%)	28 (62.2)	118 (61.5)	0.924
Type 2 diabetes, *n* (%)	12 (26.7)	62 (32.3)	0.464
COPD, *n* (%)	8 (17.8)	50 (26.0)	0.246
Cerebrovascular disease, *n* (%)	8 (17.8)	60 (31.3)	0.072
Anxiety or depression, *n* (%)	16 (35.6)	52 (27.1)	0.258

To assess the impact of frailty decompensation on adverse outcomes, multivariable logistic regression models were established for urinary incontinence, stroke, and cardiovascular events, and a Cox proportional hazards model was established for all-cause mortality, adjusting for confounders such as hemoglobin, LVEF, and NYHA class. The results showed that frailty decompensation was an independent risk factor for urinary incontinence (OR = 4.113, 95% CI [1.117–20.188], *P* = 0.049), while no significant association was found with other endpoints (Table 4).

**Table 4:** The impact of frailty decompensation on adverse outcomes.

Indicator	Stroke		Incontinence		Cardiovascular		All-cause mortality	
	OR (95% CI)	*P*	OR (95% CI)	*P*	OR (95% CI)	*P*	HR (95% CI)	*P*
Frailty decompensation	1.564 (0.618–4.03)	0.348	4.113 (1.117–20.188)	0.049	2.035 (0.794–5.305)	0.141	1.231 (0.547–2.772)	0.612
Hemoglobin (g/L)	0.985 (0.968–1.002)	0.087	1.002 (0.982–1.022)	0.873	0.982 (0.964–0.999)	0.047	0.999 (0.985–1.014)	0.926
LVEF (%)	1.013 (0.960–1.070)	0.631	0.976 (0.918–1.037)	0.429	0.99 (0.935–1.047)	0.727	0.974 (0.934–1.016)	0.224
NYHA class II	1.165 (0.421–3.335)	0.770	1.045 (0.284–4.4)	0.949	2.116 (0.749–6.075)	0.158	0.978 (0.332–2.882)	0.968
NYHA class III	1.169 (0.445–3.191)	0.754	1.113 (0.342–4.338)	0.865	1.091 (0.409–2.891)	0.860	2.388 (0.911–6.26)	0.076
NYHA class IV	1.792 (0.673–4.981)	0.249	1.625 (0.506–6.288)	0.440	1.139 (0.417–3.078)	0.797	2.211 (0.833–5.865)	0.110

### Relationship between decompensation and hospitalization frequency

Multivariable negative binomial regression analysis, adjusted for age and NYHA class, showed that frailty decompensation remained an independent risk factor for increased hospitalization frequency (IRR = 1.628, 95% CI [1.156–2.272], *P* = 0.003). Additionally, advanced age (IRR = 1.032, *P* = 0.003) and NYHA class III (IRR = 1.917, *P* = 0.005) and class IV (IRR = 2.430, *P* < 0.001) were significantly associated with higher hospitalization frequency (Table 5).

**Table 5:** Univariate and multivariable negative binomial regression analysis for the association between frailty decompensation and hospitalization frequency.

Variable	IRR (95% CI)	*P*	IRR (95% CI)	*P*
Frailty decompensation	2.326 (1.661–3.224)	<0.001	1.628 (1.156–2.272)	0.003
NYHA class				
I	Ref		Ref	
II	1.424 (0.862–2.332)	0.162	1.134 (0.686–1.858)	0.609
III	2.569 (1.61–4.047)	<0.001	1.917 (1.188–3.057)	0.005
IV	3.44 (2.169–5.383)	<0.001	2.43 (1.5–3.89)	<0.001
Age (years)	1.045 (1.021–1.07)	<0.001	1.032 (1.01–1.055)	0.003
COPD	0.746 (0.556–1.012)	0.055		
MNA-SF score	0.97 (0.916–1.026)	0.301		
Albumin (g/L)	0.991 (0.969–1.014)	0.446		
Cerebrovascular disease	1.089 (0.825–1.45)	0.553		
Hypertension	1.068 (0.819–1.387)	0.625		
Anxiety or depression	0.932 (0.705–1.242)	0.626		
Type 2 diabetes	0.946 (0.72–1.251)	0.692		
Hemoglobin (g/L)	0.998 (0.99–1.007)	0.724		
Sex	0.964 (0.745–1.25)	0.780		
BMI (kg/m^2^)	1.007 (0.956–1.061)	0.791		

## Discussion

This study addresses critical gaps in geriatric frailty research by identifying key risk factors for frailty, defining an exploratory LVEF cutoff for frailty decompensation, and quantifying the impact of decompensation on adverse clinical outcomes-all within a cohort of patients with geriatric syndromes.

The observed frailty prevalence of 44.05% is consistent with existing epidemiological data (Rodriguez-Manas & Fried, 2015). Our study not only examined the prevalence of frailty but also further elucidated its associated determinants. The independent risk factors identified-advanced age, malnutrition (lower MNA-SF scores and albumin), impaired cardiac function (lower LVEF and higher NYHA class), psychological comorbidities (anxiety/depression), and lower hemoglobin-collectively underscore the multisystem dysregulation characteristic of frailty. These findings are consistent with the conceptual framework that views physiological frailty as a consequence of dysfunction across multiple interacting organ systems  (Tromp et al., 2024; Fried et al., 2021; Wang et al., 2025). The paradoxical finding of a higher smoking prevalence in the non-frail group warrants cautious interpretation. While recent preclinical evidence suggests nicotine may influence NAD+ metabolism and aging-related pathways (Yang et al., 2023), this observation likely reflects complex confounding by factors such as survivor bias, differential comorbidities, or health behaviors, rather than indicating a protective effect of smoking. Further mechanistic and longitudinal studies are needed to clarify this relationship.

A key contribution of this study is the establishment of an LVEF cutoff of ≤51.2% for frailty decompensation, with robust diagnostic accuracy (AUC = 0.781). This threshold addresses a key limitation of previous studies by providing a standardized and objective criterion to distinguish compensated from decompensated frailty, thereby translating mechanistic insights into a potential clinical tool and fulfilling the core requirements for frailty screening proposed by an international Delphi consensus (Pedretti et al., 2025). Future multicenter cohort studies are warranted to further validate the value of this LVEF cutoff for frailty decompensation, as our clinically event-defined decompensation subgroup has demonstrated markedly worse outcomes. After adjusting for key confounders including hemoglobin, LVEF, and NYHA class, frailty decompensation remained an independent risk factor specifically for urinary incontinence. Our results demonstrate that patients with frailty decompensation have a 4.113-fold higher risk of urinary incontinence compared with non-frail individuals. Consistently, a longitudinal study based on the Chinese Health and Retirement Longitudinal Study (CHARLS) cohort reported that frail individuals had a 4.5-fold increased risk of developing urinary incontinence (Gan et al., 2025). This suggests a distinct pathophysiological link, possibly involving shared autonomic dysfunction or global physiological reserve depletion affecting pelvic floor integrity. In addition, our age-stratified correlation analyses confirm that this LVEF does not merely reflect age-related physiological decline, but rather represents an independent, physiology-based marker, further strengthening its validity. The ability of this LVEF cutoff to identify patients at risk for frailty decompensation further confirms its clinical relevance. As frailty decompensation (defined independently of LVEF) was an independent risk factor for increased hospitalization frequency, predicting both adverse health outcomes and healthcare utilization, a critical consideration in the management of complex geriatric patients  (Rosano et al., 2025).

The LVEF ≤ 51.2% threshold identified in this study may offer potential value for risk stratification and personalized intervention pending external validation. It could serve as an exploratory reference for subclinical cardiac impairment in older adults, potentially pinpointing individuals at the “compensatory edge” who may be most vulnerable to stress-induced decompensation (Abe et al., 2025; Jia et al., 2022). This exploratory, internally derived cutoff suggests a possible shift from age-based to physiology-driven care, which may help clinicians distinguish between compensated and high-risk decompensated frailty. For the latter group, it might flag the need for intensified management, including referral to multidisciplinary teams, tailored cardiac rehabilitation, meticulous medication review, and proactive advance care planning. By predicting both adverse outcomes and increased healthcare utilization, this threshold may provide a potential tool for optimizing resource allocation and implementing targeted preventive strategies in future research or clinical practice following external validation.

The predictive model for frailty progression, incorporating the identified risk and protective factors, demonstrated excellent discriminative ability (C-index = 0.84) and good calibration. This model provides a framework for identifying at-risk individuals early in the disease trajectory, enabling preventive strategies before the onset of decompensation.

Preventing frailty decompensation requires a deep understanding of its underlying mechanisms, which are intricately linked to cardiometabolic disorders. Large-scale cohort studies have unequivocally demonstrated that frailty is not only an independent risk factor for cardiovascular disease-with its coexistence with depression further amplifying this risk-but also that even in older adults without a history of cardiovascular disease, frailty and pre-frailty predict higher all-cause mortality and major adverse cardiovascular events (Damluji et al., 2021a; Zhang et al., 2025). These clinical observations are underpinned by complex, shared biological mechanisms. Central to this interplay is inflammaging, characterized by chronic low-grade inflammation. Pro-inflammatory cytokines, like IL-6, TNF-*α*, drive proteolysis, leading not only to muscle degradation and insulin resistance but also to endothelial dysfunction, atherosclerosis, and myocardial remodeling  (Santulli et al., 2025; Xu et al., 2024; Giunta, 2008; Bano et al., 2017; Kochlik et al., 2023). Meanwhile, insulin resistance, oxidative stress, and mitochondrial dysfunction form a self-perpetuating vicious cycle: chronic inflammation induces insulin resistance, while hyperglycemia exacerbates reactive oxygen species production (Mose et al., 2020; Gonçalves, Gomes & Seelaender, 2024; Rezuş et al., 2020; Mone et al., 2025; Jomova et al., 2023; Shi et al., 2024); age- or disease-related mitochondrial decline results in metabolic dysregulation, and the excessive reactive oxygen species generated further impair insulin signaling, ultimately contributing to muscle wasting, energy depletion, and metabolic imbalance (Jandeleit-Dahm et al., 2024; Luo et al., 2020; Chen et al., 2022; Khazan et al., 2018). In addition, chronic kidney disease-a common complication of cardiometabolic disorders-contributes to frailty through anemia and bone metabolism abnormalities (Steinmeyer et al., 2020; Abdu, Abdu & Arogundade, 2019; Dufour et al., 2025). Our research also demonstrated that hemoglobin is a protective factor for Frailty.

A deeper understanding of these mechanisms provides clear targets for multidimensional intervention and prevention of frailty decompensation. Current research is translating these mechanistic insights into concrete preventive and therapeutic strategies, offering new hope for breaking the vicious cycle between frailty and cardiometabolic disease. For instance, targeting core inflammatory pathways, selective IL-1*β* inhibitors have shown potential in reducing cardiovascular events, providing proof-of-concept for anti-inflammatory strategies to ameliorate frailty (Ridker et al., 2017). In the realm of mitochondrial dysfunction, agents such as urolithin A and elamipretide-which aim to enhance muscle endurance and bioenergetics-are being specifically evaluated in frail populations (Liu et al., 2022; Tung et al., 2025). In metabolic modulation, SGLT2 inhibitors have demonstrated benefits across broad populations, including frail older adults (Aldafas et al., 2024), while GLP-1 receptor agonists-despite their significant cardiometabolic advantages-require careful consideration of their potential impact on muscle mass in older individuals (Prokopidis, Daly & Suetta, 2025). In addition, hormone modulation therapies targeting common hormonal imbalances (Cao et al., 2019), and personalized medicine approaches that tailor interventions based on individual genetic, phenotypic, and biomarker profiles, constitute essential components of future intervention frameworks.

Therefore, future preventive efforts should move beyond single-factor approaches toward comprehensive, multidimensional management grounded in mechanistic understanding. By early identification and targeted intervention of the key pathways described above, it may be possible to effectively delay or even reverse frailty progression, thereby reducing cardiovascular risk and ultimately achieving healthy aging.

Several limitations must be acknowledged. The single-center design may limit generalizability, and the absence of comprehensive diastolic function assessment represents an important gap, as diastolic dysfunction is prevalent in the elderly and may contribute to frailty. Furthermore, while multiple imputation and sensitivity analyses supported the robustness of our findings, the observational nature of the study precludes causal inference. Notably, the LVEF threshold of ≤51.2% for defining frailty decompensation was derived in an exploratory, data-driven manner within a single cohort. It has not been externally validated. Before this threshold can be applied clinically, future multicenter, prospective cohort studies incorporating advanced cardiac imaging and longer follow-up are warranted to validate the LVEF cutoff and explore the causal pathways linking cardiac function to frailty decompensation and specific adverse outcomes like urinary incontinence.

## Conclusions

This study advances the understanding of frailty in geriatric syndromes by identifying key associated factors, proposing an exploratory, internally derived LVEF threshold for frailty decompensation, and demonstrating that frailty decompensation is associated with increased morbidity, mortality, and healthcare use. These findings underscore the importance of integrating cardiac function assessment into routine frailty evaluation and provide a hypothesis-generating foundation for developing targeted interventions. However, whether the LVEF threshold can be directly implemented in clinical practice requires further external validation.

##  Supplemental Information

10.7717/peerj.21514/supp-1 Data S1 Supplementary Raw data

10.7717/peerj.21514/supp-2 Fig. S1 Flow-chart of patients’ progress into the study steps

10.7717/peerj.21514/supp-3 Fig. S2 Stratified analysis of the age-LVEF correlation across different age groups (A) Scatter plots of LVEF against age within three strata: 60–74 years (*n* = 287), 75–84 years (*n* = 225), and ≥85 years (*n* = 26). Trend lines are displayed for each subgroup. (B) Bar chart summarizing the Spearman correlation coefficients (*ρ*) and the proportion of variance explained (*ρ*^2^) for each age stratum. The weak and inconsistent correlations across strata further support that age is not a major determinant of the LVEF threshold used to define frailty decompensation.

10.7717/peerj.21514/supp-4 Table S1 Multivariate Cox Regression Analysis of Frailty Risk Factors (*N* = 486)

10.7717/peerj.21514/supp-5 Supplemental Information 5 STROBE Documentation

## Additional Information and Declarations

### Data Availability

The following information was supplied regarding data availability: The raw data are available in the Supplemental File.

## References

[ref-1] Abdu A, Abdu A, Arogundade FA (2019). Prevalence and pattern of chronic kidney disease-mineral bone disorders among hemodialysis patients in Kano, northwest Nigeria. Annals of African Medicine.

[ref-2] Abe TA, Tressel W, Bartz TM, Gottdiener JS, Kamel H, Kizer JR, Longstreth Jr. WT, Shah SJ, Djoussée L, Mukamal KJ (2025). Subclinical cardiac dysfunction and circulating markers of brain injury in older adults: the cardiovascular health study. Journal of Stroke and Cerebrovascular Diseases.

[ref-3] Aldafas R, Crabtree T, Alkharaiji M, Vinogradova Y, Idris I (2024). Sodium-glucose cotransporter-2 inhibitors (SGLT2) in frail or older people with type 2 diabetes and heart failure: a systematic review and meta-analysis. Age and Ageing.

[ref-4] Álvarez-Millán L, Castillo-Castillo D, Quispe-Siccha R, Pérez-Pacheco A, Angelova M, Rivera-Sánchez J, Fossion R (2023). Frailty syndrome as a transition from compensation to decompensation: application to the biomechanical regulation of gait. International Journal of Environmental Research and Public Health.

[ref-5] Aydin AE, Altunkalem Seydi K, Gozen F, Ates Bulut E (2025). The relationship between malnutrition, frailty, and sarcopenia according to the GLIM and MNA-SF. Irish Journal of Medical Science.

[ref-6] Bano G, Trevisan C, Carraro S, Solmi M, Luchini C, Stubbs B, Manzato E, Sergi G, Veronese N (2017). Inflammation and sarcopenia: a systematic review and meta-analysis. Maturitas.

[ref-7] Barbosa da Silva A, Queiroz de Souza I, Da Silva IK, Borges Lopes Tavares da Silva M, Oliveira dos Santos AC (2020). Factors associated with frailty syndrome in older adults. Journal of Nutrition Health & Aging.

[ref-8] Cao J, Zhong MB, Toro CA, Zhang L, Cai D (2019). Endo-lysosomal pathway and ubiquitin-proteasome system dysfunction in alzheimer’s disease pathogenesis. Neuroscience Letters.

[ref-9] Chen TH, Koh KY, Lin KM, Chou CK (2022). Mitochondrial dysfunction as an underlying cause of skeletal muscle disorders. International Journal of Molecular Sciences.

[ref-10] Damluji AA, Chung SE, Xue QL, Hasan RK, Moscucci M, Forman DE, Bandeen-Roche K, Batchelor W, Walston JD, Resar JR, Gerstenblith G (2021a). Frailty and cardiovascular outcomes in the national health and aging trends study. European Heart Journal.

[ref-11] Damluji AA, Chung SE, Xue QL, Hasan RK, Walston JD, Forman DE, Bandeen-Roche K, Moscucci M, Batchelor W, Resar JR, Gerstenblith G (2021b). Physical frailty phenotype and the development of geriatric syndromes in older adults with coronary heart disease. American Journal of Medicine.

[ref-12] Dufour A, Kurtz KA, Vachey C, Mac-Way F (2025). Association between frailty and bone health in early-stage chronic kidney disease: a study from the population-based CARTaGENE cohort. Clinical Kidney Journal.

[ref-13] Fried LP, Cohen AA, Xue QL, Walston J, Bandeen-Roche K, Varadhan R (2021). The physical frailty syndrome as a transition from homeostatic symphony to cacophony. Nature Aging.

[ref-14] Fried LP, Tangen CM, Walston J, Newman AB, Hirsch C, Gottdiener J, Seeman T, Tracy R, Kop WJ, Burke G, McBurnie MA (2001). Frailty in older adults: evidence for a phenotype. Journals of Gerontology Series A: Biological Sciences and Medical Sciences.

[ref-15] Gan X, Meng L, Cao S, Bai H, Li X (2025). Impact of frailty and its change on urinary incontinence: a longitudinal analysis from two prospective studies of ageing. PLOS ONE.

[ref-16] Giunta S (2008). Exploring the complex relations between inflammation and aging (inflamm-aging): anti-inflamm-aging remodelling of inflamm-aging, from robustness to frailty. Inflammation Research.

[ref-17] Gonçalves DC, Gomes SP, Seelaender M (2024). Metabolic, inflammatory, and molecular impact of cancer cachexia on the liver. International Journal of Molecular Sciences.

[ref-18] Guidet B, De Lange DW, Boumendil A, Leaver S, Watson X, Boulanger C, Szczeklik W, Artigas A, Morandi A, Andersen F, Zafeiridis T, Jung C, Moreno R, Walther S, Oeyen S, Schefold JC, Cecconi M, Marsh B, Joannidis M, Nalapko Y, Elhadi M, Fjølner J, Flaatten H, VIP2 study group (2020). The contribution of frailty, cognition, activity of daily life and comorbidities on outcome in acutely admitted patients over 80 years in European ICUs: the VIP2 study. Intensive Care Medicine.

[ref-19] Huang W, Nurhafizah A, Frederich A, Khairunnisa AR, Kezia C, Fathoni MI, Samban S, Flindy S (2025). Risk and protective factors of poor clinical outcomes in heart failure with improved ejection fraction population: a systematic review and meta-analysis. Current Cardiology Reports.

[ref-20] Jandeleit-Dahm KAM, Kankanamalage HR, Dai A, Meister J, Lopez-Trevino S, Cooper ME, Touyz RM, Kennedy CRJ, Jha JC (2024). Endothelial NOX5 obliterates the reno-protective effect of Nox4 deletion by promoting renal fibrosis *via* activation of EMT and ROS-sensitive pathways in diabetes. Antioxidants.

[ref-21] Jia Y, Li D, Yu J, Liu Y, Li F, Li W, Zhang Q, Gao Y, Zhang W, Zeng Z, Zeng R, Liao X, Zhao Q, Wan Z (2022). Subclinical cardiovascular disease and frailty risk: the atherosclerosis risk in communities study. BMC Geriatrics.

[ref-22] Jomova K, Raptova R, Alomar SY, Alwasel SH, Nepovimova E, Kuca K, Valko M (2023). Reactive oxygen species, toxicity, oxidative stress, and antioxidants: chronic diseases and aging. Archives of Toxicology.

[ref-23] Khazan M, Hedayati M, Robati RM, Riahi SM, Nasiri S (2018). Impaired oxidative status as a potential predictor in clinical manifestations of herpes zoster. Journal of Medical Virology.

[ref-24] Kochlik B, Franz K, Henning T, Weber D, Wernitz A, Herpich C, Jannasch F, Aykaç V, Müller-Werdan U, Schulze MB, Grune T, Norman K (2023). Frailty is characterized by biomarker patterns reflecting inflammation or muscle catabolism in multi-morbid patients. Journal of Cachexia, Sarcopenia and Muscle.

[ref-25] Li N, Liu G, Gao H, Wu Q, Meng J, Wang F, Jiang S, Chen M, Xu W, Zhang Y, Wang Y, Feng Y, Liu J, Xu C, Lu H (2023). Geriatric syndromes, chronic inflammation, and advances in the management of frailty: a review with new insights. BioScience Trends.

[ref-26] Liu S, D’Amico D, Shankland E, Bhayana S, Garcia JM, Aebischer P, Rinsch C, Singh A, Marcinek DJ (2022). Effect of urolithin A supplementation on muscle endurance and mitochondrial health in older adults: a randomized clinical trial. JAMA Network Open.

[ref-27] Luo J, Mills K, Le Cessie S, Noordam R, Van Heemst D (2020). Ageing, age-related diseases and oxidative stress: what to do next?. Ageing Research Reviews.

[ref-28] Mishra M, Wu J, Kane AE, Howlett SE (2024). The intersection of frailty and metabolism. Cell Metabolism.

[ref-29] Mone P, Komici K, Guerra G, Dazzetti T, Kansakar U, Gennarelli G, Rainone A, Macina G, Di Mauro M, Iaccarino G, Testa G, Santulli G (2025). Stress hyperglycemia ratio and physical frailty in HFpEF. Cardiovascular Diabetology.

[ref-30] Mose M, Rittig N, Mikkelsen UR, Jessen N, Bengtsen MB, Christensen B, Jørgensen JOL, Møller N (2020). A model mimicking catabolic inflammatory disease: a controlled randomized study in humans. PLOS ONE.

[ref-31] Muszalik M, Gurtowski M, Doroszkiewicz H, Gobbens RJ, Kędziora-Kornatowska K (2019). Assessment of the relationship between frailty syndrome and the nutritional status of older patients. Clinical Interventions in Aging.

[ref-32] Pandey A, Kitzman D, Reeves G (2019). Frailty is intertwined with heart failure: mechanisms, prevalence, prognosis, assessment, and management. JACC: Heart Failure.

[ref-33] Pedretti RFE, Asteggiano R, Gevaert AB, Bowen TS, Caselli S, Cornelissen VA, Christodorescu R, Derosa G, Dievart F, Kurpas D, Osto E, Richter D, Semb AG, Steca P, Guasti L, Ferrini M (2025). Cardiovascular risk factors management in older adults: a clinical consensus statement from the European Association of Preventive Cardiology of the ESC and the ESC Council for Cardiology Practice. European Journal of Preventive Cardiology.

[ref-34] Picca A, Coelho-Junior HJ, Calvani R, Marzetti E, Vetrano DL (2022). Biomarkers shared by frailty and sarcopenia in older adults: a systematic review and meta-analysis. Ageing Research Reviews.

[ref-35] Prokopidis K, Daly RM, Suetta C (2025). Weighing the risk of GLP-1 treatment in older adults: should we be concerned about sarcopenic obesity?. Journal of Nutrition Health & Aging.

[ref-36] Rezuş E, Burlui A, Cardoneanu A, Rezuş C, Codreanu C, Pârvu M, Rusu Zota G, Tamba BI (2020). Inactivity and skeletal muscle metabolism: a vicious cycle in old age. International Journal of Molecular Sciences.

[ref-37] Ridker PM, Everett BM, Thuren T, MacFadyen JG, Chang WH, Ballantyne C, Fonseca F, Nicolau J, Koenig W, Anker SD, Kastelein JJP, Cornel JH, Pais P, Pella D, Genest J, Cifkova R, Lorenzatti A, Forster T, Kobalava Z, Vida-Simiti L, Flather M, Shimokawa H, Ogawa H, Dellborg M, Rossi PRF, Troquay RPT, Libby P, Glynn RJ, CANTOS Trial Group (2017). Antiinflammatory therapy with canakinumab for atherosclerotic disease. New England Journal of Medicine.

[ref-38] Rizza S, Morabito P, De Meo L, Farcomeni A, Testorio G, Cardellini M, Ballanti M, Davato F, Pecchioli C, Di Cola G, Mavilio M, Federici M (2021). IL-6 levels influence 3-month all-cause mortality in frail hospitalized older patients. Aging and Disease.

[ref-39] Rodriguez-Manas L, Fried LP (2015). Frailty in the clinical scenario. Lancet.

[ref-40] Rosano GMC, Teerlink JR, Kinugawa K, Bayes-Genis A, Chioncel O, Fang J, Greenberg B, Ibrahim NE, Imamura T, Inomata T, Kuwahara K, Moura B, Onwuanyi A, Sato N, Savarese G, Sakata Y, Sweitzer N, Wilcox J, Yamamoto K, Metra M, Coats AJS (2025). The use of left ventricular ejection fraction in the diagnosis and management of heart failure: a clinical consensus statement of the Heart Failure Association (HFA) of the ESC, the Heart Failure Society of America (HFSA), and the Japanese Heart Failure Society (JHFS). European Journal of Heart Failure.

[ref-41] Santulli G, Sabatelli G, Wang B, Savino M, Bruno FP, Jankauskas SS, Massaro A, Peluso C, Vicario M, Savino L, Varzideh F, D’Onghia ML, Mone P (2025). Interplay between frailty and cardiometabolic disorders: from pathophysiology to clinical implications. Cardiovascular Diabetology.

[ref-42] Shi TF, Zhou Z, Jiang WJ, Huang TL, Si JQ, Li L (2024). Hyperglycemia-induced oxidative stress exacerbates mitochondrial apoptosis damage to cochlear stria vascularis pericytes *via* the ROS-mediated Bcl-2/CytC/AIF pathway. Redox Report.

[ref-43] Soysal P, Stubbs B, Lucato P, Luchini C, Solmi M, Peluso R, Sergi G, Isik AT, Manzato E, Maggi S, Maggio M, Prina AM, Cosco TD, Wu YT, Veronese N (2016). Inflammation and frailty in the elderly: a systematic review and meta-analysis. Ageing Research Reviews.

[ref-44] Steinmeyer Z, Delpierre C, Soriano G, Steinmeyer A, Ysebaert L, Balardy L, Sourdet S (2020). Hemoglobin concentration: a pathway to frailty. BMC Geriatrics.

[ref-45] Thillainadesan J, Scott IA, Le Couteur DG (2020). Frailty, a multisystem ageing syndrome. Age and Ageing.

[ref-46] Tromp J, Kosiborod MN, Angermann CE, Collins SP, Teerlink JR, Ponikowski P, Biegus J, Ferreira JP, Nassif ME, Psotka MA, Brueckmann M, Blatchford JP, Steubl D, Voors AA (2024). Treatment effects of empagliflozin in hospitalized heart failure patients across the range of left ventricular ejection fraction: results from the EMPULSE trial. European Journal of Heart Failure.

[ref-47] Tung C, Varzideh F, Farroni E, Mone P, Kansakar U, Jankauskas SS, Santulli G (2025). Elamipretide: a review of its structure, mechanism of action, and therapeutic potential. International Journal of Molecular Sciences.

[ref-48] Wang S, Li Q, Hu J, Chen Q, Wang S, Xue QL, Huang C, Sun H, Liu M (2025). Association of multimorbidity patterns and order of physical frailty and cognitive impairment occurrence: a prospective cohort study. Age and Ageing.

[ref-49] Xu Y, Mao T, Wang Y, Qi X, Zhao W, Chen H, Zhang C, Li X (2024). Effect of gut microbiota-mediated tryptophan metabolism on inflammaging in frailty and sarcopenia. Journals of Gerontology Series A: Biological Sciences and Medical Sciences.

[ref-50] Yang L, Shen J, Liu C, Kuang Z, Tang Y, Qian Z, Guan M, Yang Y, Zhan Y, Li N, Li X (2023). Nicotine rebalances NAD+ homeostasis and improves aging-related symptoms in male mice by enhancing NAMPT activity. Nature Communications.

[ref-51] Zeng P, Li M, Cao J, Zeng L, Jiang C, Lin F (2024). Association of metabolic syndrome severity with frailty progression among Chinese middle and old-aged adults: a longitudinal study. Cardiovascular Diabetology.

[ref-52] Zhang Z, Xu H, Zhang R, Yan Y, Ling X, Meng Y, Zhang X, Wang Y (2025). Frailty and depressive symptoms in relation to cardiovascular disease risk in middle-aged and older adults. Nature Communications.

