# Supplementary figures and images for: Analysis of influencing factors for frailty in geriatric syndrome patients and the impact of frailty decompensation on major adverse events
Source: PeerJ. 2026 Jul 14;14:e21514. doi: 10.7717/peerj.21514 (PMC13378463; doi:10.7717/peerj.21514)

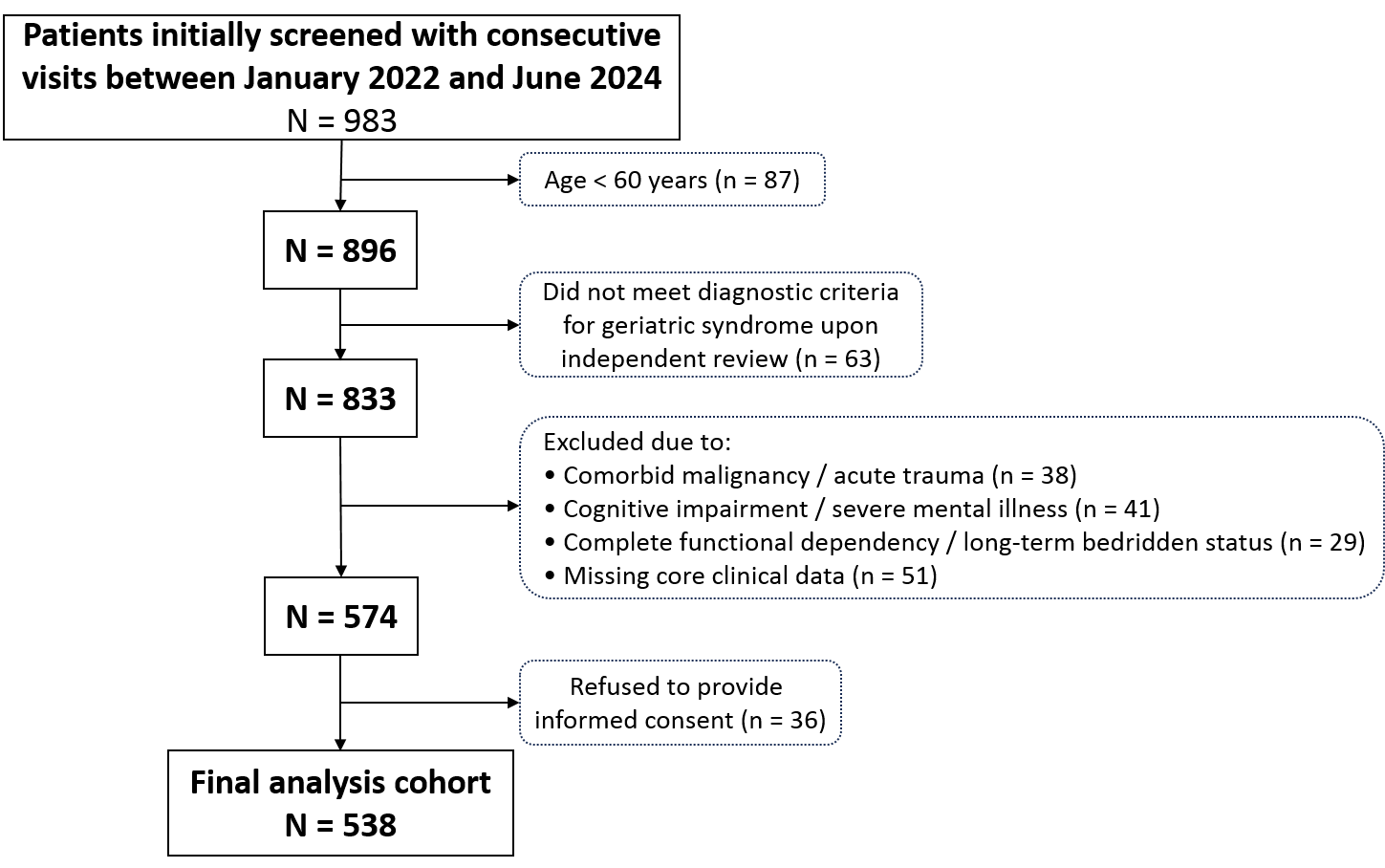

Supplement: Fig. S1 [file peerj-14-21514-s002.png]

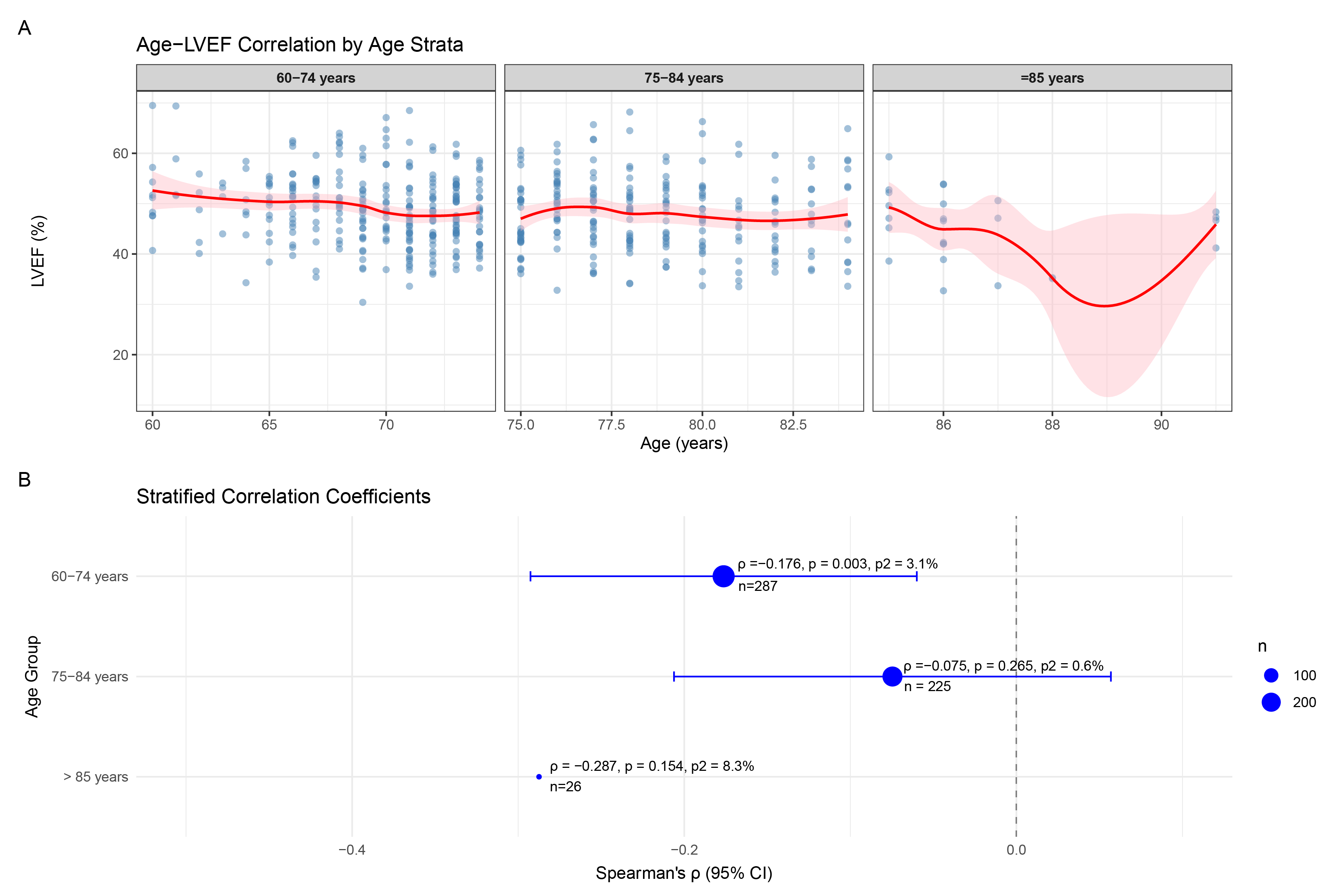

Supplement: Fig. S2 — (A) Scatter plots of LVEF against age within three strata: 60–74 years (n = 287), 75–84 years (n = 225), and ≥85 years (n = 26). Trend lines are displayed for each subgroup. (B) Bar chart summarizing the Spearman correlation coefficients (ρ) and the proportion of variance explained (ρ2) for each age stratum. The weak and inconsistent correlations across strata further support that age is not a major determinant of the LVEF threshold used to define frailty decompensation. [file peerj-14-21514-s003.png]
