# Supplementary material for: Analysis of influencing factors for frailty in geriatric syndrome patients and the impact of frailty decompensation on major adverse events
Source: PeerJ. 2026 Jul 14;14:e21514. doi: 10.7717/peerj.21514 (PMC13378463; doi:10.7717/peerj.21514)
Supplement: Table S1 [file peerj-14-21514-s004.docx]

**Table S1 Multivariate Cox Regression Analysis of Frailty Risk Factors (N = 486)**

| **Variable** | **HR (95% CI)** | ***P*** |
| --- | --- | --- |
| Age(years) | 1.112 (1.083-1.143) | **< 0.001** |
| MNA SF score | 0.811 (0.756-0.871) | **< 0.001** |
| LVEF (%) | 0.957 (0.935-0.979) | **< 0.001** |
| Albumin (g/L) | 0.951 (0.928-0.975) | **< 0.001** |
| Hemoglobin (g/L) | 0.98 (0.97-0.989) | **< 0.001** |
| Serum Creatinine (umol/L) | 1.002 (0.994-1.011) | 0.591 |
| NYHA_class2 | 1.87 (0.971-3.602) | 0.061 |
| NYHA_class3 | 5.411 (2.937-9.969) | **< 0.001** |
| NYHA_class4 | 5.801 (3.11-10.819) | **< 0.001** |
| BMI (kg/m2) | 0.931 (0.879-0.986) | **0.015** |
| Smoking | 0.879 (0.629-1.228) | 0.449 |
| Hypertension | 1.001 (0.734-1.366) | 0.995 |
| Cerebrovascular disease | 0.879 (0.624-1.236) | 0.458 |
| Anxiety or Depression | 2.288 (1.636-3.2) | **< 0.001** |
