# Supplementary material for: Analysis of influencing factors for frailty in geriatric syndrome patients and the impact of frailty decompensation on major adverse events
Source: PeerJ. 2026 Jul 14;14:e21514. doi: 10.7717/peerj.21514 (PMC13378463; doi:10.7717/peerj.21514)
Supplement: Supplemental Information 5 [file peerj-14-21514-s005.docx]

STROBE Statement—checklist of items that should be included in reports of observational studies

|  | Item No. | Recommendation | Page  No. | Relevant text from manuscript |
| --- | --- | --- | --- | --- |
| **Title and abstract** | 1 | (*a*) Indicate the study’s design with a commonly used term in the title or the abstract | 1 | Title: Analysis of Influencing Factors on... and the impact of... The abstract begins by stating that the research is on frailty in elderly patients with major adverse events. The methods section clearly mentions "prospective cohort study". |
|  |  | (*b*) Provide in the abstract an informative and balanced summary of what was done and what was found | 1 | Background: The research aims to identify the risk factors for frailty progression in elderly syndrome patients, construct a predictive model, and evaluate the impact of heart function-related frailty decompensation on adverse events. Methods: From January 2022 to June 2024, 538 patients were included and grouped according to the Fried phenotype criteria. The LVEF threshold was determined using the ROC curve, and data were analyzed through multivariate Cox regression, χ² test, etc. Results: 44.05% of the patients were in a frail state. Six independent risk factors were identified, and LVEF ≤ 52.5% was the best threshold for frailty decompensation. The decompensation group had a higher incidence of adverse events. Conclusion: The high incidence of frailty in elderly syndrome patients is emphasized, and early identification of decompensation can improve prognosis. |
| Introduction | | | |  |
| Background/rationale | 2 | Explain the scientific background and rationale for the investigation being reported | 1,2 | Background: Frailty syndrome (FS) is a state in the elderly population characterized by the cumulative decline of multiple organ systems, leading to a decrease in physiological reserves and weakened stress resistance. It is prone to adverse outcomes such as falls and hospitalizations. Currently, there is a lack of research on the differences in adverse outcomes between the decompensated and compensated subgroups of frailty, and there is no unified diagnostic standard for decompensation. Principle: Existing studies mostly focus on metabolic or molecular mechanisms, lacking empirical evidence on the application of cardiac function (such as LVEF) in the decompensation of frailty. It is necessary to identify risk factors, construct a predictive model including LVEF, and determine the threshold for decompensation to fill the clinical gap. |
| Objectives | 3 | State specific objectives, including any prespecified hypotheses | 3 | Objective 1: To identify the independent risk factors for frailty in elderly patients with syndromes; Objective 2: To construct a predictive model for the progression of frailty incorporating left ventricular ejection fraction (LVEF); Objective 3: To determine LVEF ≤ 52.5% as the criterion for frailty decompensation through ROC curve, and compare the incidence of major adverse events (all-cause mortality, stroke, cardiovascular events, urinary incontinence) between the compensated (LVEF > 52.5%) and decompensated subgroups; Objective 4: To explore the association between frailty decompensation and hospitalization frequency. |
| Methods | | | |  |
| Study design | 4 | Present key elements of study design early in the paper | 3 | This study was a prospective cohort study conducted at Chuzhou Hospital Affiliated to Anhui Medical University. Elderly patients with geriatric syndrome from January 2022 to June 2024 were included and classified into the frailty group and the non-frailty group based on the Fried phenotype criteria. The frailty group was further divided into the compensated subgroup and the decompensated subgroup according to the LVEF threshold. The median follow-up time was 24 months. Data were analyzed using multivariate Cox regression, ROC analysis, and other methods. |
| Setting | 5 | Describe the setting, locations, and relevant dates, including periods of recruitment, exposure, follow-up, and data collection | 3 | Setting: Department of Geriatrics and Department of Cardiology, Chuzhou Hospital Affiliated to Anhui Medical University; Location: Chuzhou City, Anhui Province (Postal Code 239000); Date: Recruitment period from January 2022 to June 2024, median follow-up period of 24 months (interquartile range 18-30 months), data collection throughout the recruitment and follow-up process, including baseline demographic information, laboratory and imaging index tests, frailty assessment, etc. |
| Participants | 6 | (*a*) *Cohort study*—Give the eligibility criteria, and the sources and methods of selection of participants. Describe methods of follow-up  *Case-control study*—Give the eligibility criteria, and the sources and methods of case ascertainment and control selection. Give the rationale for the choice of cases and controls  *Cross-sectional study*—Give the eligibility criteria, and the sources and methods of selection of participants | 3 | Inclusion criteria: age ≥ 60 years old, clinical symptoms, electrocardiogram and echocardiogram results meeting the diagnostic criteria for geriatric syndrome; exclusion criteria: concurrent malignant tumors, hematological diseases, acute trauma, cognitive impairment, dementia, severe mental illness, complete functional dependence or bedridden, severe hearing impairment or communication disorders; source: patients visiting the Geriatrics Department and Cardiology Department of Chuzhou Hospital Affiliated to Anhui Medical University; selection method: consecutive inclusion of patients meeting the criteria; follow-up method: regular outpatient follow-up every 3 months, monthly telephone follow-up, and recording of adverse event occurrence. |
|  |  | (*b*) *Cohort study*—For matched studies, give matching criteria and number of exposed and unexposed  *Case-control study*—For matched studies, give matching criteria and the number of controls per case | 1,4 | This study was a non-matched cohort study. According to the Fried phenotype criteria, 538 patients were divided into the frailty group (237 cases) and the non-frailty group (301 cases). The frailty group was further divided into the decompensated subgroup (108 cases) and the compensated subgroup (129 cases) based on LVEF ≤ 52.5% (determined by the ROC curve). |
| Variables | 7 | Clearly define all outcomes, exposures, predictors, potential confounders, and effect modifiers. Give diagnostic criteria, if applicable | 4 | Outcome variables: Major adverse events (all-cause mortality, stroke, cardiovascular events, urinary incontinence), frequency of hospitalization; Exposure variable: Left ventricular ejection fraction (LVEF); Predictors: Age, Mini Nutritional Assessment Short Form (MNA-SF) score, serum albumin level, New York Heart Association (NYHA) functional class, anxiety or depressive state, etc.; Potential confounding factors: Gender, smoking history, drinking history, BMI, comorbidities (type 2 diabetes, hypertension, COPD, cerebrovascular disease); Diagnostic criteria: Frailty was defined based on the Fried phenotype criteria (meeting ≥ 3 of the 5 indicators: unintentional weight loss > 4.5 kg within 1 year, self-reported fatigue, reduced physical activity, decreased grip strength, slow gait speed); All-cause mortality was verified by medical records or family reports; Stroke was confirmed by head CT or MRI; Cardiovascular events (acute myocardial infarction, heart failure hospitalization, clinically significant arrhythmia) were diagnosed by the attending physician; Urinary incontinence was defined as involuntary leakage of urine at least once a week, as reported by the patient or caregiver and confirmed by the nurse; Anxiety or depression was assessed using the Hamilton Depression Scale (HAMD). |
| Data sources/ measurement | 8* | For each variable of interest, give sources of data and details of methods of assessment (measurement). Describe comparability of assessment methods if there is more than one group | *4* | *Data sources: Demographic information, smoking and drinking history, BMI, NYHA heart function classification, comorbidities, etc. were obtained through medical record review and patient interviews; MNA-SF scores were completed through scale interviews; HAMD scores were evaluated using standardized scales; LVEF was detected by echocardiography; serum albumin levels were detected by laboratory biochemical tests; adverse events were obtained through follow-up records. Details of the assessment methods: LVEF was measured using the apical biplane Simpson method recommended by the American Society of Echocardiography in 2015, performed by two physicians with 8 and 10 years of experience in geriatric cardiac imaging and certified at level 3 by the Chinese Society of Echocardiography, using the GE Vivid E95 echocardiography system software (Version 2023.1) for analysis. The calculation method was (end-diastolic volume - end-systolic volume) / end-diastolic volume × 100%, and intra-observer and inter-observer consistency analyses were conducted (ICC values were 0.93 and 0.89, respectively); serum albumin was detected by an automatic biochemical analyzer; comparability: All patients' LVEF detection and scale evaluations were conducted using uniform standards and instruments to ensure consistent assessment methods between groups.* |
| Bias | 9 | Describe any efforts to address potential sources of bias | 4,5 | 1. Selection bias: By continuously enrolling patients who meet the criteria, selection bias is reduced.  2. Measurement bias: LVEF testing is conducted by two experienced and certified physicians, and consistency analysis is performed to ensure measurement accuracy; standardized testing methods are used for laboratory indicators such as serum albumin; unified instructions are provided for scale assessment (MNA-SF, HAMD).  3. Attrition bias: Regular outpatient follow-ups and monthly phone calls are combined to reduce the attrition rate.  4. Confounding bias: In the multivariate Cox regression analysis, potential confounding factors such as gender, smoking history, drinking history, BMI, and comorbidities are adjusted.  5. Missing data bias: Missing data (<5%) are handled using multiple imputation (10 imputations). |
| Study size | 10 | Explain how the study size was arrived at | None | This study did not elaborate on the sample size calculation method. Based on the consecutive clinical visits from January 2022 to June 2024 and patients meeting the inclusion and exclusion criteria, a total of 538 cases were included. The sample size can meet the basic requirements for multivariate analysis and subgroup comparisons. |

Continued on next page

| Quantitative variables | 11 | Explain how quantitative variables were handled in the analyses. If applicable, describe which groupings were chosen and why | 5 | Quantitative variable processing: Quantitative variables that conform to a normal distribution (such as age, MNA-SF score, serum albumin level, and LVEF) are expressed as mean ± standard deviation, and the comparison between groups is conducted using the independent sample t-test; non-normally distributed quantitative variables are expressed as median (interquartile range), and the comparison between groups is conducted using the Mann-Whitney U test. Grouping method: LVEF is treated as a quantitative variable, and the optimal cut-off value (52.5%) is determined through ROC curve analysis. The frailty group is then divided into decompensated (LVEF ≤ 52.5%) and compensated (LVEF > 52.5%) subgroups. The reason for this is to clearly define the objective cardiac function threshold for decompensated frailty to achieve clinically operable risk stratification. |
| --- | --- | --- | --- | --- |
| Statistical methods | 12 | (*a*) Describe all statistical methods, including those used to control for confounding | 5 | Statistical software: SPSS 26.0; Descriptive statistics: Quantitative variables were expressed as mean ± standard deviation or median (interquartile range) according to the distribution type, and categorical variables were expressed as frequency (percentage); Group comparison: Independent sample t-test was used for normally distributed quantitative variables, Mann-Whitney U test for non-normally distributed ones, and χ² test for categorical variables; Risk factor analysis: Univariate Cox regression was used to screen potential predictive factors (P < 0.10), and multivariate Cox regression was used to analyze independent risk factors, controlling for confounding factors such as gender, smoking history, drinking history, BMI, and comorbidities. Hazard ratio (HR) and 95% confidence interval (CI) were reported; ROC curve analysis was used to determine the optimal cut-off value of LVEF, and the area under the curve (AUC), sensitivity, and specificity were calculated; Correlation analysis: Spearman rank correlation analysis was used to analyze the association between hospitalization frequency and decompensation of frailty. |
|  |  | (*b*) Describe any methods used to examine subgroups and interactions | None | This study did not detail the specific methods for subgroup analysis and interaction tests. It only mentioned that the frailty group was divided into compensated and decompensated subgroups to compare the incidence of adverse events. |
|  |  | (*c*) Explain how missing data were addressed | 5 | The proportion of missing data is less than 5%. Multiple imputation method (10 imputations) is adopted for processing. Meanwhile, sensitivity analysis is conducted by excluding the imputed data to verify the robustness of the results. |
|  |  | (*d*) *Cohort study*—If applicable, explain how loss to follow-up was addressed  *Case-control study*—If applicable, explain how matching of cases and controls was addressed  *Cross-sectional study*—If applicable, describe analytical methods taking account of sampling strategy | None | By combining regular outpatient follow-ups (every three months) with monthly phone follow-ups, the rate of loss to follow-up was minimized to the greatest extent possible, but no specific strategy for handling the data of those lost to follow-up was clearly stated. |
|  |  | (*e*) Describe any sensitivity analyses | 5,6 | Sensitivity analysis was conducted by excluding imputed data (complete case analysis, n = 511) and comparing the results with those including imputed data (HR difference < 5%) to verify the robustness of the independent risk factors. The results remained statistically significant, such as age (HR = 1.168 vs 1.176) and LVEF (HR = 0.919 vs 0.915). |
| Results | | | | |
| Participants | 13* | (a) Report numbers of individuals at each stage of study—eg numbers potentially eligible, examined for eligibility, confirmed eligible, included in the study, completing follow-up, and analysed | 3,5,6 | Potential qualified candidates: The specific number is not clear; eligible candidates: 538 patients continuously included; researchers included: 538 cases (no cases excluded as eligible); completed follow-up: The number of those lost to follow-up is not clear; median follow-up time: 24 months; number of analysts: 538 cases (using multiple imputation to fill in missing data, complete case analysis: 511 cases) |
|  |  | (b) Give reasons for non-participation at each stage | None | Those not meeting the inclusion criteria are directly excluded. |
|  |  | (c) Consider use of a flow diagram | None | All the details of the research subjects that met the inclusion criteria in this study have been clearly described in the text. |
| Descriptive data | 14* | (a) Give characteristics of study participants (eg demographic, clinical, social) and information on exposures and potential confounders | 5,6 | Research subject characteristics: Age (75.41 ± 5.72 years in the frail group, 73.35 ± 6.53 years in the non-frail group), gender (no significant difference between the two groups), smoking history (no significant difference), alcohol consumption history (no significant difference), BMI (no specific numerical value reported, no significant difference), MNA-SF score (11.36 ± 2.07 in the frail group, 11.77 ± 2.49 in the non-frail group), serum albumin (41.35 ± 7.43 g/L in the frail group, 42.78 ± 6.41 g/L in the non-frail group), LVEF (45.24 ± 6.79% in the frail group, 48.70 ± 6.17% in the non-frail group), NYHA III-IV grade (55.70% in the frail group, 46.84% in the non-frail group), anxiety or depression (24.47% in the frail group, 16.94% in the non-frail group), comorbidities (type 2 diabetes, hypertension, COPD, cerebrovascular disease, no details of differences between groups reported) |
|  |  | (b) Indicate number of participants with missing data for each variable of interest | 5 | All variable missing data account for less than 5%, and the specific number of missing values for each individual variable is not reported. |
|  |  | (c) *Cohort study*—Summarise follow-up time (eg, average and total amount) | 3,5,6 | The median follow-up period was 24 months (interquartile range: 18 - 30 months), and the average follow-up time and total follow-up time were not reported. |
| Outcome data | 15* | *Cohort study*—Report numbers of outcome events or summary measures over time | *7* | *Number of outcome events (decompensation subgroup vs compensated subgroup): Urinary incontinence (30.34% vs 16.89%), all-cause mortality (43.82% vs 23.65%), stroke (48.31% vs 33.11%), cardiovascular events (64.04% vs 44.59%); The distribution of outcome events at different time points was not reported.* |
|  |  | *Case-control study—*Report numbers in each exposure category, or summary measures of exposure | *4,5,6,7* | *This study was designed as a prospective cohort study (not a case-control study), and thus the data were not presented in the standard format of "number of exposure categories" or "summary indicators of exposure" as in a case-control study. However, based on the relevant content of the core exposure variable (left ventricular ejection fraction, LVEF) in the cohort study, corresponding information on distribution, grouping, and summary was provided.* |
|  |  | *Cross-sectional study—*Report numbers of outcome events or summary measures | *5,7* | *This study was designed as a prospective cohort study (not a cross-sectional study), so the data were not presented in accordance with the standard framework of "outcome event quantity or summary indicators" for cross-sectional studies. However, this article has extracted the core quantitative and summary information related to "outcome events" in the cohort study, and has correspondingly explained the contents of the report.* |
| Main results | 16 | (*a*) Give unadjusted estimates and, if applicable, confounder-adjusted estimates and their precision (eg, 95% confidence interval). Make clear which confounders were adjusted for and why they were included | 6 | Unadjusted estimates: Single-factor Cox regression was used to screen potential predictive factors (P < 0.10), and specific HR values were not reported; Adjusted estimates (independent risk factors): Age (HR = 1.176, 95% CI: 1.065 - 1.299, P = 0.001), MNA-SF score (HR = 0.732, 95% CI: 0.584 - 0.918, P = 0.007), serum albumin (HR = 0.904, 95% CI: 0.844 - 0.968, P = 0.004), LVEF (HR = 0.915, 95% CI: 0.878 - 0.954, P < 0.001), NYHA III-IV grade (HR = 3.285, 95% CI: 1.698 - 6.353, P < 0.001), anxiety or depression (HR = 1.548, 95% CI: 1.093 - 2.192, P = 0.014); Reasons for adjustment: These factors were related to the progression of frailty in the univariate analysis and might affect the results as confounding factors, so they were included in the multivariate model for adjustment. |
|  |  | (*b*) Report category boundaries when continuous variables were categorized | 7 | The grouping boundaries for the continuous variable LVEF were set at 52.5% (determined by the ROC curve, AUC = 0.852, sensitivity = 0.805, specificity = 0.835). Based on this, the frailty group was divided into decompensated (≤ 52.5%) and compensated (> 52.5%) subgroups; the grouping boundaries for the frequency of hospitalization: 1-5 times, 6-10 times, > 10 times. |
|  |  | (*c*) If relevant, consider translating estimates of relative risk into absolute risk for a meaningful time period | None | - |

Continued on next page

| Other analyses | 17 | Report other analyses done—eg analyses of subgroups and interactions, and sensitivity analyses | 5,6 | Subgroup analysis: The frailty group was divided into compensated and decompensated subgroups based on LVEF, and the incidence of adverse events and hospitalization frequency were compared; Sensitivity analysis: Completely case analysis was conducted by excluding missing data to verify the robustness of the independent risk factor results; No interaction analysis results were reported |
| --- | --- | --- | --- | --- |
| Discussion | | | | |
| Key results | 18 | Summarise key results with reference to study objectives | 7,8 | Key Result 1: The prevalence of frailty among elderly syndrome patients was 44.05%. Advanced age, low MNA-SF score, low serum albumin, low LVEF, NYHA III-IV grade, anxiety or depression were independent risk factors for the progression of frailty. Key Result 2: LVEF ≤ 52.5% was the optimal threshold for frailty decompensation (AUC = 0.852). Key Result 3: The incidence and frequency of all-cause mortality, stroke, cardiovascular events, urinary incontinence, and hospitalization in the decompensation subgroup were significantly higher than those in the compensated subgroup (Spearman correlation coefficient = 0.620, P < 0.001), which was consistent with the purpose of the study, namely identifying risk factors, determining the threshold for decompensation, and exploring its clinical impact. |
| Limitations | 19 | Discuss limitations of the study, taking into account sources of potential bias or imprecision. Discuss both direction and magnitude of any potential bias | 8 | Limitation 1: This is a single-center study, and the sample source is limited to Chuzhou Hospital Affiliated to Anhui Medical University. This may affect the generalizability of the results. Limitation 2: The diastolic function was not comprehensively evaluated, and abnormal diastolic function may be related to frailty, which may miss potential influencing factors. Limitation 3: The basis for sample size calculation was not detailed, and there may be a risk of insufficient statistical power. Limitation 4: The dropout rate and reasons for dropout were not clearly defined, which may introduce dropout bias. Potential bias direction: The single-center sample may overestimate the risk factor effects of a specific population. The absence of diastolic function assessment may underestimate the impact of cardiac function on frailty. If dropout is related to the outcome, it may lead to result bias (for example, if the dropout patients are mostly those with good outcomes, it may overestimate the incidence of adverse events). |
| Interpretation | 20 | Give a cautious overall interpretation of results considering objectives, limitations, multiplicity of analyses, results from similar studies, and other relevant evidence | 7,8 | The risk factors for frailty identified in this study (such as advanced age, malnutrition, abnormal cardiac function, and mental illness) are consistent with the existing meta-analyses and consensus. The threshold of LVEF ≤ 52.5% for decompensation supplements the clinical diagnostic criteria, and the association between decompensation and adverse outcomes is in line with the prognosis pattern of patients with heart failure. At the same time, the limitations of a single-center study need to be considered, and the results need to be verified by multi-center studies. Further research is needed to explore the impact of diastolic function. The results were not over-interpreted due to multiple analyses (such as multivariate regression and subgroup comparisons), and it is emphasized that a comprehensive judgment should be made based on other evidence. |
| Generalisability | 21 | Discuss the generalisability (external validity) of the study results | 8 | The research subjects were elderly patients with syndrome from a single hospital in Anhui Province of China, with specific regional and clinical characteristics (such as a higher proportion of patients with cardiac function problems). The results may not be applicable to other regions, other medical institutions, or elderly populations without clear cardiac function abnormalities; Extrapolation-based suggestions: In the future, multi-center and diverse population studies (such as from different regions and with different disease spectra) need to be conducted to verify the applicability of risk factors and LVEF thresholds, in order to enhance the extrapolation ability of the results. |
| Other information | |  | | |
| Funding | 22 | Give the source of funding and the role of the funders for the present study and, if applicable, for the original study on which the present article is based |  | Anhui Provincial Natural Science Foundation (Project Number: 2022e07020019); Role of the funder: Not involved in research design, data collection, analysis and interpretation, as well as manuscript writing and submission decisions. |

*Give information separately for cases and controls in case-control studies and, if applicable, for exposed and unexposed groups in cohort and cross-sectional studies.

**Note:** An Explanation and Elaboration article discusses each checklist item and gives methodological background and published examples of transparent reporting. The STROBE checklist is best used in conjunction with this article (freely available on the Web sites of PLoS Medicine at http://www.plosmedicine.org/, Annals of Internal Medicine at http://www.annals.org/, and Epidemiology at http://www.epidem.com/). Information on the STROBE Initiative is available at www.strobe-statement.org.
